# Anomalous Nernst Effect in Flexible Co-Based Amorphous Ribbons

**DOI:** 10.3390/s23031420

**Published:** 2023-01-27

**Authors:** Marcio A. Correa, Armando Ferreira, Arthur L. R. Souza, João. M. Dantas Neto, Felipe Bohn, Filipe Vaz, Galina V. Kurlyandskaya

**Affiliations:** 1 Departamento de Física, Universidade Federal do Rio Grande do Norte, Natal 59078-900, RN, Brazil; 2 Centro de Física das Universidades do Minho e do Porto (CF-UM-UP), Universidade do Minho, 4710-057 Braga, Portugal; 3 LaPMET—Laboratório de Física para Materiais e Tecnologias Emergentes, Universidade do Minho, 4710-057 Braga, Portugal; 4 Department of Magnetism and Magnetic Nanomaterials, Ural Federal University, 620002 Ekaterinburg, Russia; 5 Department of Electricity and Electronics, University of the Basque Country UPV/EHU, 48940 Leioa, Biscay, Spain

**Keywords:** co-based amorphous ribbon, anomalous Nernst effect, magnetic sensors, magnetic properties, magnetization process, flexible sensors

## Abstract

Fe${}_{3}$Co${}_{67}$Cr${}_{3}$Si${}_{15}$B${}_{12}$ ribbons with a high degree of flexibility and excellent corrosion stability were produced by rapid quenching technique. Their structural, magnetic, and thermomagnetic (Anomalous Nernst Effect) properties were studied both in an as-quenched (NR) state and after stress annealing during 1 h at the temperature of 350 °C and a specific load of 230 MPa (AR). X-ray diffraction was used to verify the structural characteristics of our ribbons. Static magnetic properties were explored by inductive technique and vibrating sample magnetometry. The thermomagnetic curves investigated through the Anomalous Nernst Effect are consistent with the obtained magnetization results, presenting a linear response in the thermomagnetic signal, an interesting feature for sensor applications. Additionally, Anomalous Nernst Effect coefficient $S_{ANE}$ values of 2.66μV/K and 1.93μV/K were estimated for the as-quenched and annealed ribbons, respectively. The interplay of the low magnetostrictive properties, soft magnetic behavior, linearity of the thermomagnetic response, and flexibility of these ribbons place them as promising systems to probe curved surfaces and propose multifunctional devices, including magnetic field-specialized sensors.

## 1. Introduction

Green processes and advanced energy saving technologies are crucial requests nowadays [1,2,3,4,5,6]. Among them, physical and electrophysical techniques employing no or a small amount of water/organic solvents have become very important [7,8]. In this scenario, the development of highly efficient sensors with reduced energy consumption levels becomes essential for the future [9,10,11,12]. For instance, one of the tendencies is the miniaturization of sensor devices, allowing to save both energy and the amount of employed materials [13,14]. Although a wide range of physical and chemical phenomena has been explored for sensor applications in recent years, special attention was paid to the materials which can demonstrate the ability to be employed in multifunctional devices, as well as materials having stable properties with respect to special environmental conditions. For instance, the change in the electrical resistance of nanostructures has a great appeal for temperature, gas, and humidity sensors [15,16,17,18]. Furthermore, changes in electrical voltage can be used for magnetic and electrical field sensors [19,20,21,22], including magnetic field sensors working in principle on the magnetoimpedance effect (GMI) [23,24,25,26,27] and being one of the most efficient types of the sensors for obtaining the high sensitivity for room temperature applications.

Another key point to mention with respect to the active research directions is the significant increase in demand for multifunctional sensors with flexible properties [26,27]. Within this context, the sensors in which the measurements of distinct physical/chemical properties with flexible sensitive elements allow the miniaturization of the devices and the production of sensors adjustable to curved surfaces. The last feature is very important for many biomedical applications (including sports medicine) where an adjustable body or particular shape design is highly desired.

The thermomagnetic effects arise as an exciting alternative to developing multifunctional flexible sensors. Phenomena such as Anomalous Nernst (ANE) [28,29,30,31] and Longitudinal Spin-Seebeck (LSSE) effects [32,33,34,35] can be easily accessed through a slight thermal gradient (∇T) applied to a ferromagnetic material. Usually, thermomagnetic effects are studied in ferromagnetic nanostructures (thin films) grown onto rigid substrates [35,36]. The geometry of these materials permits the adjustment of the ferromagnetic properties during the film deposition up to the desired, making possible the tuning of the thermomagnetic response. However, the employment of a flexible element, in turn, provides a path for modifications of the magnetic properties, especially in the case of magnetic materials having high magnetostriction coefficient [30]. A possible alternative to address this challenge is the use of flexible materials without substrates, such as amorphous ribbons or even ribbon-based composites [37,38]. For instance, previously, a number of studies were devoted to the design of GMI composites consisting of rapidly quenched magnetic ribbons, with thin-film coverings deposited by sputtering deposition [39,40]. For sensor applications, flexible properties related to the change of shape are requested together with soft magnetic properties. In this case, the most magnetically soft ribbons having nanocrystalline structures become not appropriate due to their poor mechanical properties and very limited flexibility [41,42].

Surprisingly, the number of studies of thermomagnetic effects as ANE in amorphous ribbons is scarce [43,44]. The main reason for the reduced interest relies on the difficulties of obtaining ferromagnetic ribbons with magnetic properties favorable for thermomagnetic applications. In this case, properties such as high magnetic permeability (μ), high saturation magnetization ($M_{s}$), low coercive field ($H_{c}$), and low magnetostriction properties can hardly be reached simultaneously. Nonetheless, in a recent study, Kurlyandskaya and co-workers [45] have brought to light a ferromagnetic system with remarkable potential. Specifically, the authors have produced Co-based ferromagnetic flexible ribbons with soft magnetic properties and very small negative magnetostriction constant, and high corrosion stability. These features allow reaching ribbon with interesting linearity in the magnetization dependence concern to the external applied magnetic field at the low field interval. These ribbons demonstrated excellent potential for high-frequency sensor applications, explored by using magnetoimpedance effect [45]. From now, new studies about the potential application for thermomagnetic voltage generation can improve the applicability of the alloy. The interplay of these properties is an excellent starting point for ANE studies and, consequently, the development of multifunctional flexible sensor applications.

It is worth mentioning that Fe${}_{3}$Co${}_{67}$Cr${}_{3}$Si${}_{15}$B${}_{12}$ amorphous ribbons are well-studied materials with a number of interesting properties [46], showing the possibility of modification of the effective magnetic anisotropy by heat treatments, in certain conditions appearance the second-order magnetic anisotropy, and characterized by excellent corrosion stability requested in biological applications [47]. Moreover, Chromium (Cr) doping results in complex changes in the physical properties of the ribbon. Cr addition allows verifying an increase in corrosion stability, which leads to a better quality of the free side of the ribbon in comparison with the composition without Cr doping. Magnetically, the ribbon presents a very stable response in the induced magnetic anisotropy induction, which is most important for the technological applications requiring an external magnetic field of a few Oersted (Oe) [45].

In this work, the structural, magnetic, and thermomagnetic properties, specifically the Anomalous Nernst Effect, were studied in the amorphous CoFeCrSiB rapidly quenched ribbons, with very small negative magnetostriction constant, both in as-quenched state and after stress annealing during 1 h at the temperature of 350 °C and a specific load of 230 MPa.

## 2. Materials and Methods

**Figure 1:**
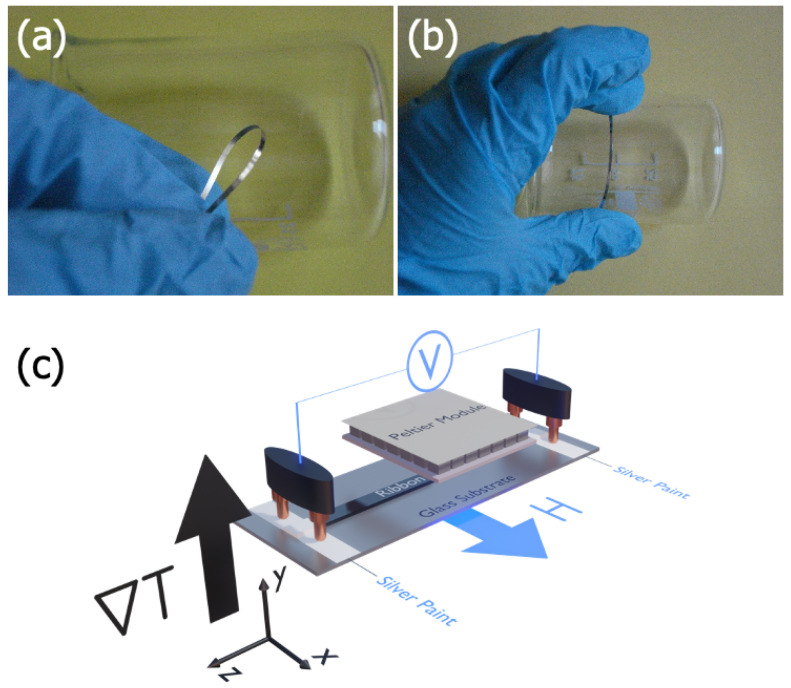
General view of the Fe${}_{3}$Co${}_{67}$Cr${}_{3}$Si${}_{15}$B${}_{12}$ amorphous ribbons of the 45 mm length showing the high degree of flexibility; (**a**) ribbon arranged in a circle (**b**) ribbon placed onto the cylindrical surface of 50 mm in diameter. (**c**) Schematic representation of the experimental setup for Anomalous Nernst Effect measurements. The ribbon was connected to a glass substrate with silver paint cured for 24 h. The Peltier module was responsible for the thermal gradient ($\vec{\nabla }T$) perpendicular to the ribbon plane. Spring gold contacts were used for the thermomagnetic measurements, while an external magnetic field was generated by an electromagnet.

Fe${}_{3}$Co${}_{67}$Cr${}_{3}$Si${}_{15}$B${}_{12}$ ribbons were produced by rapid quenching technique onto Cu rotating weal in air conditions [45,48]. The ribbons were obtained as large batches of the order of 10 m. Their dimensions were width of ≈ 0.8 mm and thickness of $t_{R}\approx 0.24\;\mu$m. The lengths varied for different experimental studies, being 35 mm for inductive hysteresis loop measurements, 5 mm for vibrating sample magnetometry (VSM), and 12 mm for ANE measurements. It is important to point out that the ribbon length used for VSM and ANE measurements must be similar once the ANE curves mimic the magnetization one for a given field alignment. Ribbons demonstrated excellent flexibility and can be curved in accordance with the shape of different surfaces, as depicted in Figure 1a,b. The alloy has a Curie temperature close to 170 °C, a crystallization temperature of 570 °C, a saturation magnetization of $M_{s}=365$ G, and a very low negative value of saturation magnetostriction constant, $\lambda_{s}\approx -10^{-7}$. The structural, magnetic, and thermomagnetic properties were studied both in an as-quenched (NR) state and after stress annealing for 1 h at the temperature of 350 °C and a specific load of 230 MPa (AR). The structural features of the ribbons were obtained through X-ray diffraction (XRD) studies by using a Rigaku diffractometer model MiniFlex (Rigaku, Tokyo, Japan), operating with Cu-k${}_{\alpha }$ radiation in θ−2θ configuration.

Inductive magnetic hysteresis loops were measured using a homemade conventional inductive system. Angular dependence of the magnetic properties was investigated via magnetization curves measured using a VSM LakeShore Model 7404 (VSM, Lake Shore 7404, Westerville, OH, USA). Depending on the particular application, the size of the ribbon element can vary significantly from a few mm to tens of cm [49,50]. The samples with variations in length differ from each other due to a change in the demagnetizing factor, affecting the value of the estimated value of effective magnetic anisotropy. For observation of the magnetic domain structure, magneto-optical Kerr (MOKE) microscopy (Evico, Dresden, Germany) was used. Although the quasi-static magnetic behavior of the ribbons has already been discussed in published articles [49,50], it is essential to ensure that the same properties were obtained. Therefore, these characterizations were done once the thermomagnetic and the magnetic properties are connected to each other.

For the Anomalous Nernst Effect measurements, we employed a homemade system, as depicted in Figure 1c. In this setup, the ribbon was connected to a glass substrate through silver paint to make easier the electrical contacts during the measurements, as well as to isolate electrically the ribbon from the heat sink, which was a metallic block in our case. To improve the thermal stability of our system, a high thermal conductive graze is used between the ribbon and the substrate. Although interface effects can be present in our heterostructure (ribbon + substrate), the thicknesses of the ribbons allow us to ensure that the ANE signal is mainly from the bulk signal of the samples. A micro-Peltier module was responsible for the $\vec{\nabla }T$ applied perpendicular to the ribbon plane. The temperature difference between the top of the ribbon and the bottom of the glass substrate (ΔT) was measured by using micro-thermocouples. The temperature control is done through currents in the micro-Peltier module. Although a rigorous experimental procedure is performed to ensure similar ΔT values for distinct samples, small differences can still be observed. The external magnetic field was oriented in the plane of the sample for distinct $\varphi_{H}$ orientations with respect to the electrical contact direction. The alignment shown in Figure 1c is defined as $\varphi_{H}$ in a similar way taken for the magnetization curves. In particular, we define the angular direction $\varphi_{H}=0^{{}^{\circ}}$ for $\vec{H}$ transverse to the main axis of the ribbon, while we assume $\varphi_{H}=90^{{}^{\circ}}$ for $\vec{H}$ along the main axis of the sample. The measurements were carried out by varying the current in the Peltier module, which allowed us to control ΔT. The thermomagnetic signal, i.e., the induced voltage, was measured using a high-resolution multimeter Keithley 2700 series.

## 3. Results and Discussion

### 3.1. Structural and Magnetic Characterization

**Figure 2:**
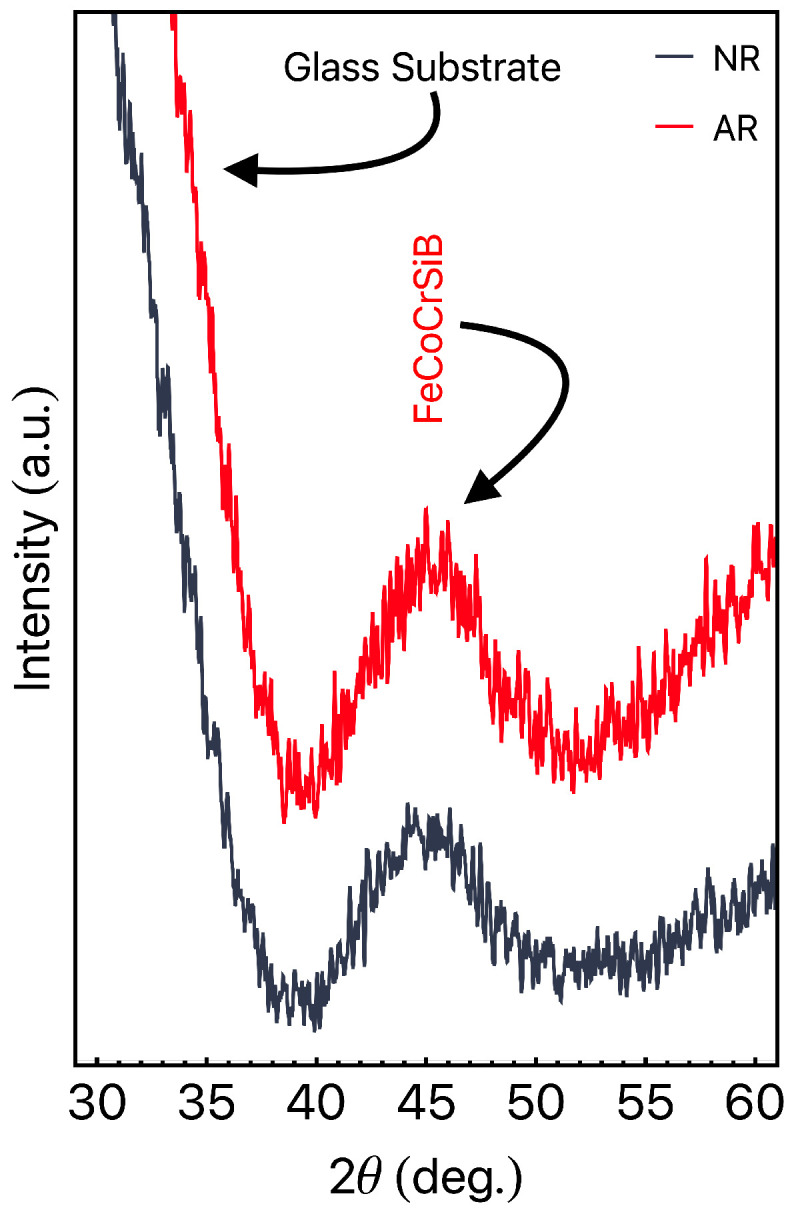
X-ray diffraction results for the NR and AR ribbons. The measurements were performed with the ribbons onto glass substrates without the use of silver paint.

Figure 2 depicts the XRD results for the NR and AR ribbons. From the analysis of the results, the amorphous character of both samples becomes clear. This feature is confirmed by the broad and low-intensity peak located at $2\theta \approx 45^{{}^{\circ}}$, which is associated with Co-based alloys. The other broad peak having higher intensity is related to the amorphous glass substrate. It is important to point out that, even with the annealing performed in the AR ribbon, the amorphous structure remains unchanged for this material. During the stress annealing, three experimental parameters come into play: the temperature, the stress intensity, and the time. In our case, the temperature of 350 °C and low values of stress were considered, ensuring the soft magnetic behavior and linear shape of the magnetization curves at a low field regime.

The very soft magnetic properties are interesting features for flexible sensor applications once the ribbon can be used to cover curved surfaces and be submitted to a small thermal gradient. Our structural findings are in accordance with that one observed in the literature for similar ribbons [45].

**Figure 3:**
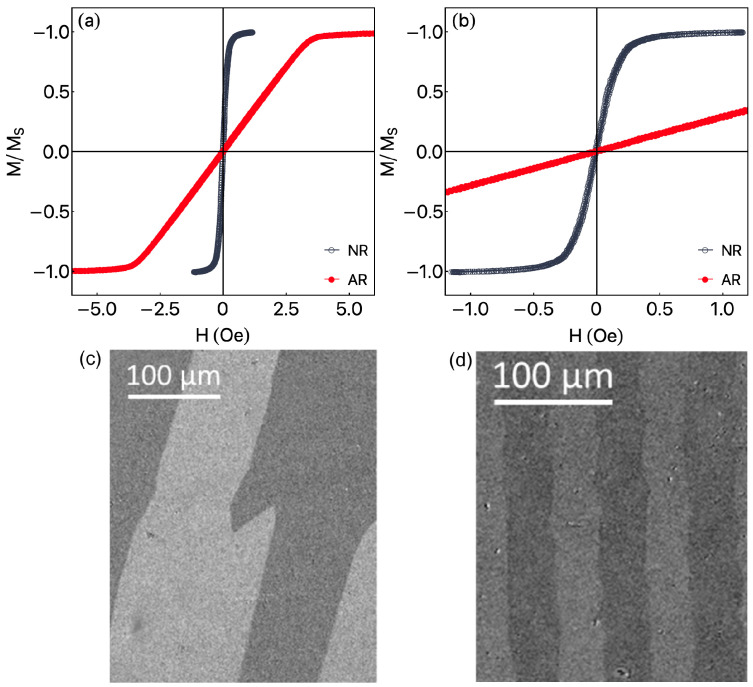
(**a**) Inductive hysteresis loops of amorphous Fe${}_{3}$Co${}_{67}$Cr${}_{3}$Si${}_{15}$B${}_{12}$ ribbons of 35 mm lengths in initial state NR and after stress-annealing AR for the magnetic field applied along the long axis of the ribbon. (**b**) Detailed view of the curves for a small magnetic field range. (**c**) Magnetic domains image for near zero fields for NR ribbon, in which the long axis of the ribbon is vertical. (**d**) Magnetic domains image for AR ribbon, in which the long axis of the ribbon is horizontal.

Figure 3a,b shows the inductive magnetic hysteresis loops, obtained by standard inductive technique, for the external magnetic field applied along the long axis of the ribbon. For the NR ribbon, although the shape of the magnetization curves can be described as an S-loop, there is a long interval in the small magnetic fields for which the linear dependence of M(H) is well fitted. The AR ribbon, in turn, has negligible coercivity and a clear linear dependence of magnetization on the applied magnetic field up to the anisotropy field of the ribbon. Here we can definitely observe the difference in magnetic behavior for NR and AR ribbons. The results for the NR one suggest an effective magnetic anisotropy having a major longitudinal component, while the ones for the AR ribbon undoubtedly indicate a transverse effective magnetic anisotropy. In this case, the induced anisotropy and the shape anisotropy are competing with each other. As aforementioned, the ribbons of such composition have very small negative saturation magnetostriction constant, $\lambda_{s}\approx -10^{-7}$, and, therefore, they can be used for stress annealing and formation of the transverse induced magnetic anisotropy [45].

The surface magnetic domains confirm our interpretation and are in agreement with the results of magnetic measurements and previous results reported in the literature. These measurements were done for a near zero external magnetic field, i.e., with the samples in the remanent state. Specifically, Figure 3c discloses tilted domains, primarily orientated along the main axis of the ribbon. Figure 3d shows typical zig-zag surface domains, a result of the stress annealing of Co-based ribbons, leading to a transverse magnetic anisotropy, which can be connected to the Fe–Fe or Cr–Cr pair ordering [51,52,53] as well as the non-uniform distribution of the internal stress. As a result, the induced anisotropy axis is oriented along the width of the ribbon [47,54]. It is known that the in-plane component of the magnetization should be oriented perpendicular to the direction of the applied stress [47]. After stress annealing, the domains are generally bounded by 180° domains walls complicated by a zig-zag sub-structure: the alternating of the tilted domain boundaries is due to an additional out-of-plane anisotropy, which leads to the structure of the observed domain at low field presented in Figure 3d.

Regarding the magnetization process, given the field is along the main axis in the inductive experiment, although some domain wall motion is expected for the NR ribbon, for both, the changes of magnetization are especially connected with coherent rotations of the magnetization of the domains with little, if any, hysteresis, a fact assigned by the very soft magnetic properties of the samples.

**Figure 4:**
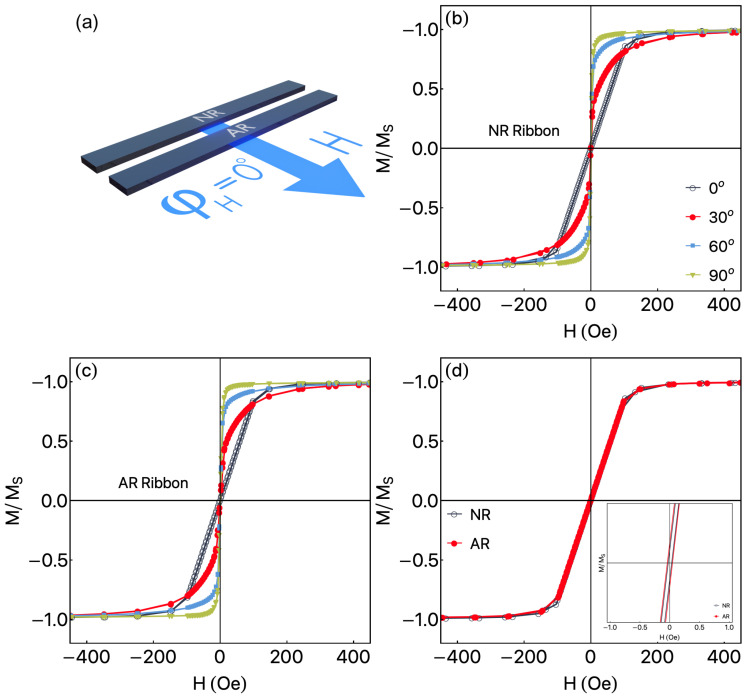
Magnetization curves for NR and AR ribbons. (**a**) Definition of $\varphi_{H}$, in which $\varphi_{H}=0^{{}^{\circ}}$ is assumed to be when the magnetic field is perpendicular to the main axis of the ribbon. (**b**) Normalized magnetization curves for the NR ribbon, measured at distinct $\varphi_{H}$ values. (**c**) Similar plots for AR ribbon. (**d**) NR and AR magnetization curves measured for $\varphi_{H}=0^{{}^{\circ}}$, showing the mirroring between the results. Inset depicts the low field curve, showing the coercive field of our ribbons.

Figure 4 shows the angular magnetization curves measured by VSM, once these properties are an interesting feature for the ANE characterization. It is worth remarking that we define $\varphi_{H}=0^{{}^{\circ}}$ as the orientation when the external magnetic field is perpendicular to the main axis of the ribbon, as we can see in Figure 4a. The external magnetic field is always oriented in the plane of the ribbon, in a sense, $\varphi_{H}=90^{{}^{\circ}}$ takes place when $\vec{H}$ is parallel to the main axis of the sample.

Figure 4b shows the magnetization curves for the NR ribbon. Strong dependence of the magnetization curves is observed as the $\varphi_{H}$ increases. Considering the dimensions of the ribbon, the results may be interpreted in terms of strong shape anisotropy. For $\varphi_{H}=0^{{}^{\circ}}$ a magnetization behavior with a tiny coercive field, $H_{c}\approx 2$ Oe, and saturation field $H_{s}$ of around 220 Oe is observed. However, the most remarkable feature is related to the linearity of the curves at low magnetic fields, ±80 Oe, for the measurements performed at $\varphi_{H}=0^{{}^{\circ}}$. Similar behavior was verified for the AR ribbon, as we can see in Figure 4c. Again, the shape anisotropy dominates in such a way that makes the magnetic properties of the NR and AR ribbons to be very similar, with a tiny increase in the coercive field, $H_{c}\approx 8$ Oe. The shape anisotropy commands the magnetization curves for distinct $\varphi_{H}$ values. For the curves measured for $\varphi_{H}=0^{{}^{\circ}}$ the linearity of the magnetization is reproduced. To compare the NR and AR ribbons, Figure 4d shows the magnetization curves for $\varphi_{H}=0^{{}^{\circ}}$. From this figure, it is evident the very same behavior for the two ribbons. Inset depicts the very low coercive field of our ribbons. The comparison of the remarkable difference between the magnetic behavior of short and long ribbons presented in Figure 3 and Figure 4 can be understood by the contribution of the demagnetizing fields of the short samples.

In both cases of NR and AR ribbons, the samples present soft magnetic behavior, with magnetization curves having very small $H_{c}$, irrespectively of $\varphi_{H}$ measured curves. This latter feature can also be interpreted as a feature of the small influence of the magnetostrictive properties of the Co-based alloy on the magnetic ones.

The transition from the longitudinal to transverse effective anisotropy (depicted in Figure 3), as well as the dependence of such evolution with the dimensions of the ribbons (Figure 3 and Figure 4) are issues that deserve further studies. Anyway, the magnetic properties of this alloy present an interesting behavior for low magnetic field sensor applications. It is important to point out that these properties can be reproduced in other magnetic phenomena. Therefore, from now, we show the thermomagnetic response, measured using ANE.

### 3.2. Anomalous Nernst Effect

The Anomalous Nernst Effect is the generation of an electrical field $\vec{E}$ when a ferromagnetic material with magnetization $\vec{M}$ is submitted to a thermal gradient $\vec{\nabla }T$. The induced electrical field is described through the relation [28]

(1) $${\vec{E}}_{ANE}=-S_{ANE}\left(\hat{M}\times \vec{\nabla }T\right),$$

with $\hat{M}$ being the unit vector associated with the orientation of $\vec{M}$. Here, $S_{ANE}$ is related with the ANE coefficient $\lambda_{ANE}$ by

(2) $$\lambda_{ANE}=\frac{S_{ANE}}{\mu_{{}^{\circ}}M_{s}},$$

where $\mu_{{}^{\circ}}$ is the vacuum magnetic permeability, and $M_{s}$ is the saturation magnetization of the studied material. Experimentally, we are able to measure an ANE voltage $V_{ANE}$ through contacts put on top of the sample, distant *L* one each other. Such voltage is given by

(3) $$V_{ANE}=-\int_{0}^{L}{\vec{E}}_{ANE}\cdot d\vec{l}.$$

Hence, from Equations (1) and (3), one can notice it is possible to handle the V${}_{ANE}$ by changing the magnetic state of the ribbon, i.e., $\vec{M}$. Moreover, the $V_{ANE}$ signal is strongly dependent on the angular measurements between the generated ${\vec{E}}_{ANE}$, magnetization $\vec{M}$, and the direction of the electric contacts $\vec{L}$. This feature can be explored to produce directional magnetic sensor devices since changes in the $\vec{M}$ intensity and direction can produce distinct thermomagnetic $V_{ANE}$ responses.

Considering the flexible state of the ribbons, as well as the absence of effects devoted to magnetostriction, ANE measurements can be interesting to employ amorphous ribbons to generate electric signals in systems that require curved sensory covers.

**Figure 5:**
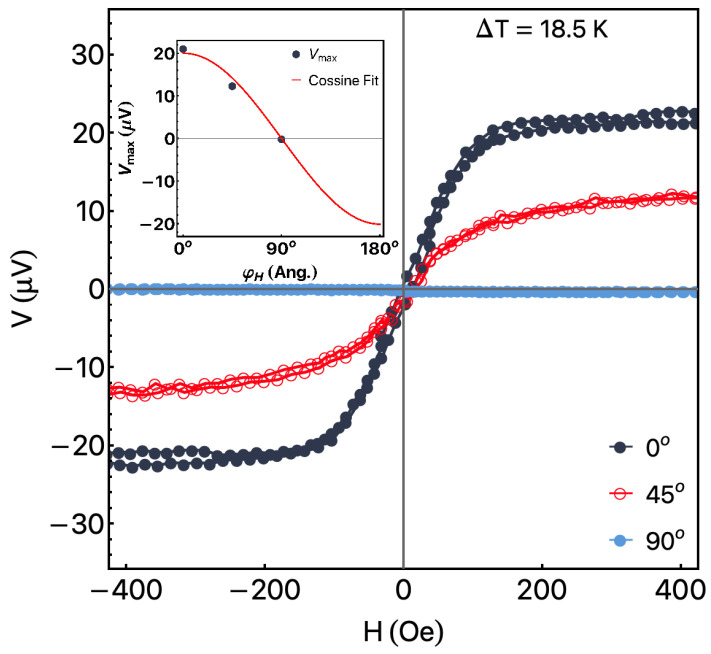
Angular dependence of the $V_{max}$ as a function of *H* for the AR ribbon measured at ΔT=18.5 K. The inset shows $V_{max}$ as a function of the $\varphi_{H}$ values. The red solid line is a fit of the experimental data using a cosine function.

Figure 5 shows the angular dependence of the ANE curves for AR ribbon measured at ΔT=18.5 K. As expected, the increase in $\varphi_{H}$ leads to a decrease in the ANE signal. This feature is connected to the rotation of the $\vec{E}$ with respect to $\vec{L}$ (voltage detection direction defined by the contacts). For $\varphi_{H}=90^{{}^{\circ}}$, we observe the absence of ANE response. As we can observe in the inset, the reduction of $V_{max}$ with $\varphi_{H}$ does not present a linear behavior. Instead of it, from Equations (1) and (3), we notice the dependence is well described by a cosine function, as confirmed. It is important to point out that the ANE response for the NR ribbon presents similar results, which corroborate with the correct assembly of the experimental apparatus.

**Figure 6:**
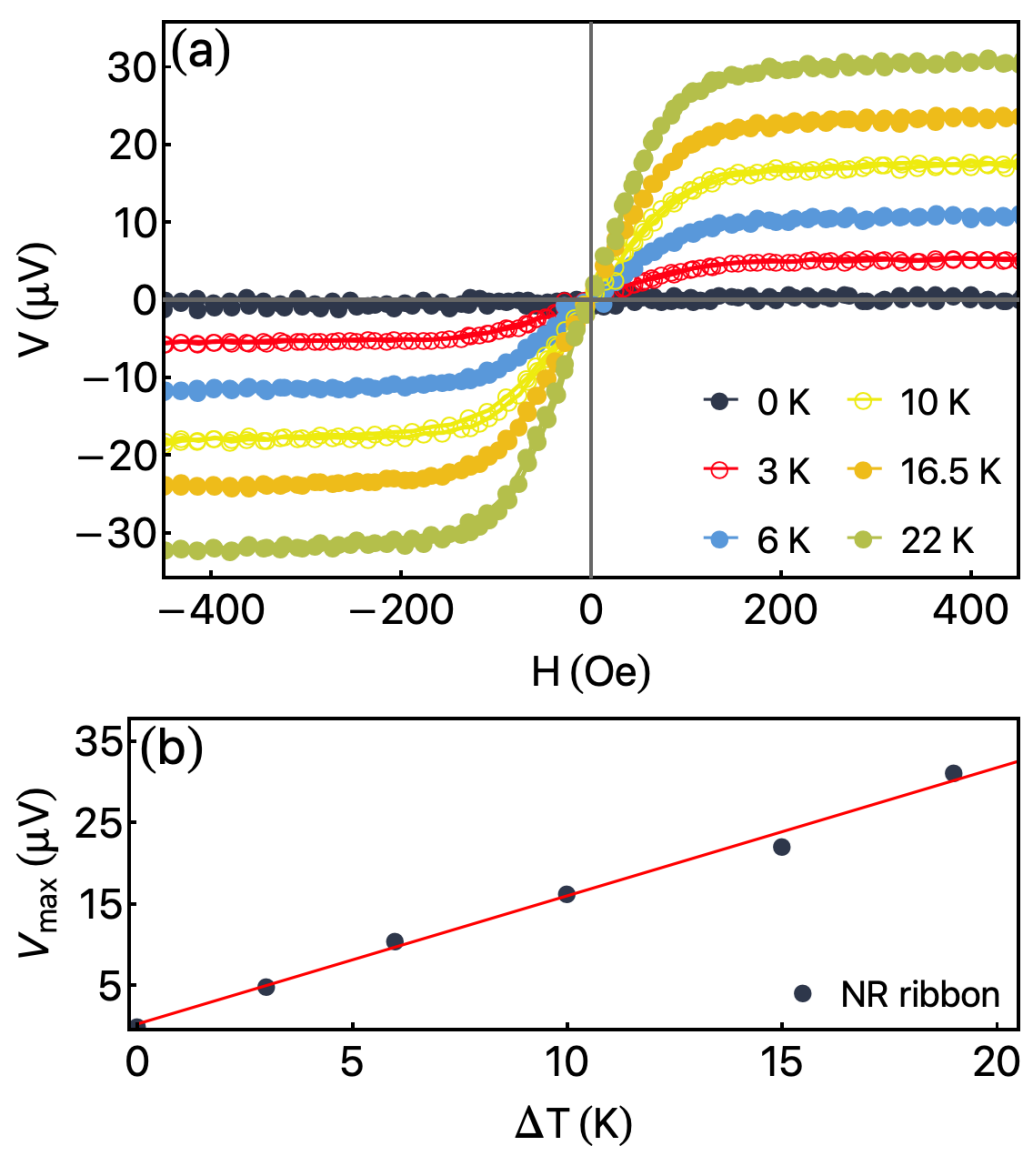
Anomalous Nernst Effect measurement for NR ribbon. (**a**) ANE voltage as a function of external magnetic field for distinct ΔT values. (**b**) $V_{max}$ as a function of ΔT for NR ribbon. From the slope of this curve, we estimated the $S_{ANE}$ value of our sample.

Figure 6 shows the ANE response for the NR ribbon. The measurements were performed as a function of the external magnetic field for distinct ΔT values at $\varphi_{H}=0^{{}^{\circ}}$. In particular, ΔT=0 K indicates that the temperatures on top of the ribbons and bottom of the glass substrate are the same. Moreover, in our experiment, we heat the top of the ribbon while the bottom of the glass substrate is in thermal contact with a heat sink. For this field’s configuration, the electrical field $\vec{E}$ is along the detection direction $\vec{L}$ defined by the electrical contacts. Consequently, for ΔT≠0 K, the shape of thermomagnetic curves mirrors the magnetization one, while for ΔT=0 K, we observe the absence of ANE voltage. The increase in ΔT leads to a linear increase in the maximum value reached by the thermomagnetic curves, named here as $V_{max}$. Taking $V_{max}$ as a function of ΔT, it is possible to estimate $S_{ANE}$. Experimentally, the $S_{ANE}$ value can be calculated through [55,56]

(4) $$S_{ANE}=\frac{V_{max}t_{R}}{L\Delta T_{R}},$$

where $\Delta T_{R}$ is the temperature difference in the ribbon, which is related to ΔT through

(5) $$\Delta T_{R}=\frac{t_{R}K_{s}}{t_{s}K_{R}}\Delta T,$$

in which $K_{s}$ and $K_{R}$ are the thermal conductivity of the substrate (glass) and of the ribbon, respectively, and $t_{s}$ and $t_{R}$ are the thickness of the substrate and ribbon.

Figure 6b presents $V_{max}$ as a function of ΔT for the NR ribbon. First, it is possible to confirm the linear behavior of the experimental results, a feature expected for the ANE effect. Second, considering $K_{s}=1.15$ W/mK and $t_{s}=0.15$ mm for the substrate, and $K_{R}=133.79$ W/mK and $t_{R}=0.24$ μm for the ribbon, from Equations (4) and (5) we achieve $S_{ANE}=2.66\mu$ V/K for the NR ribbon. This value demonstrates how efficient the conversion of thermal gradient on electrical voltage $V_{ANE}$ for this system.

**Figure 7:**
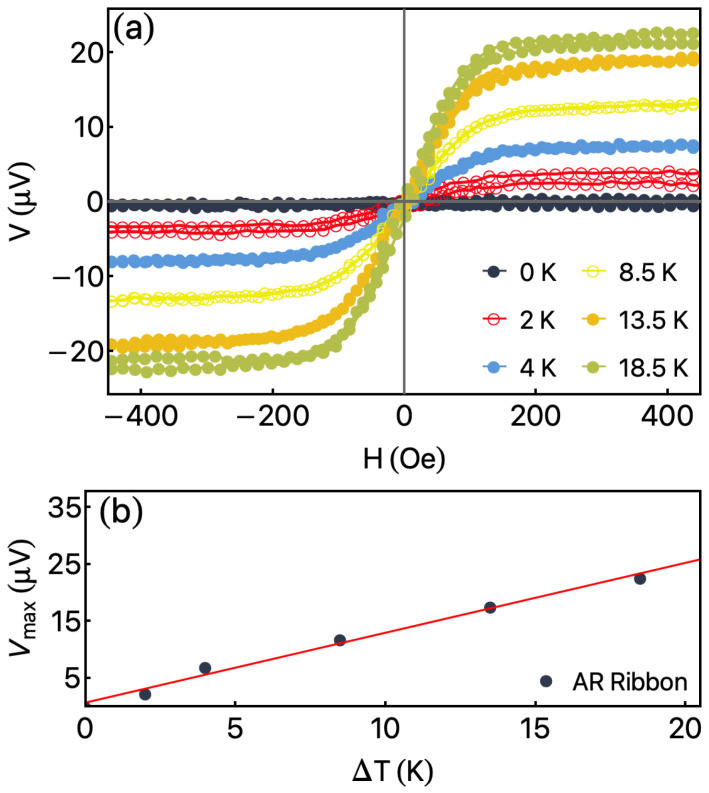
Anomalous Nernst Effect measurement for AR ribbon. (**a**) ANE voltage as a function of external magnetic field for distinct ΔT values. (**b**) $V_{max}$ as a function of ΔT for AR ribbon.

Figure 7, in turn, shows the ANE response for the AR ribbon. In Figure 7a, the curves were obtained as a function of the external magnetic field, with $\varphi_{H}=0^{{}^{\circ}}$, for distinct ΔT values. Again, the alignment between the $\vec{M}$, $\vec{E}$, and $\vec{L}$ allow us to mirror the shape of the magnetization curves in the ANE response. For ΔT=0 K, we observe the absence of the thermomagnetic voltage, as expected [see Equation (1)]. The increase in ΔT discloses a raise of $V_{max}$, without modifications in the shape of the curves.

The most striking finding resides in the linearity of the ANE curve at low field values found for the AR ribbon as well. This feature is a consequence of the low magnetostrictive properties of the alloy. As observed, after the annealing, the magnetization properties and the ANE response do not change considerably. Despite it, after the annealing, the main modification is associated with the electrical conductivity of the ribbon, which causes changes in the $V_{max}$ values.

From the slope of $V_{max}$ as a function of the ΔT, depicted in Figure 7b, the $S_{ANE}$ can be estimated using the Equations (4) and (5). Taking into account the same values previously mentioned, here we estimated $S_{ANE}=1.93\;\mu$V/K. This value is smaller than that of one observer for the NR ribbon, a feature associated with the modification in the electrical resistivity of the ribbon after the annealing. The reached $S_{ANE}$ for our flexible Co-based ribbons are compared with similar systems produced in thin films deposited onto rigid substrates [57,58].

**Figure 8:**
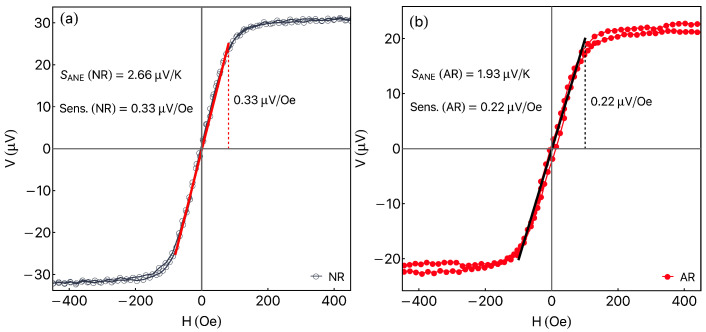
(**a**) Anomalous Nernst Effect response for the NR ribbon as a function of the magnetic field for ΔT=22 K. The red line is the linear fit between ±80 Oe where the thermomagnetic response presents a linear behavior. From the fitting, it is possible to calculate the ANE sensitivity of 0.33μV/Oe for the NR ribbon. (**b**) A similar plot for the AR ribbon measured at ΔT=18.5 K, revealing a sensitivity of 0.22μV/Oe for the AR ribbon.

As discussed before, the ANE has a great appeal for green energy generation. The exploit of such effect in flexible magnetic systems shows interesting applicability for ribbons. In our case, in addition to the ANE signal, the ribbons present a very interesting linearity at a low magnetic field range, which can be explored as magnetic field sensors. Within this context, Figure 8a depicts representative ANE response for NR ribbon as a function of the magnetic field measured with ΔT=22 K. Taking a linear fit between ±80 Oe (red line), it is possible to calculate the ANE sensitivity (Sens.). For the NR ribbon, we obtain Sens${}_{NR}=0.33\;\mu$V/Oe. Considering a similar procedure for the AR ribbon, Figure 8b shows the result measured at ΔT=18.5 K, for which we achieve Sens${}_{AR}=0.22\;\mu$V/Oe. It is important to point out that the ANE sensitivity is strongly dependent on the measured ΔT. However, experimentally, this parameter is easily controlled Peltier modulus.

The observed difference between Sens${}_{NR}$ and Sens${}_{AR}$ values still requires additional studies opening the possibilities of future research. For example, the existing techniques of structural investigation of nanocrystalline materials with stress-induced magnetic anisotropy anisotropic stress distribution were observed, indicating an important role of anisotropic stress distribution in the stress-induced magnetic anisotropy formation [59]. In the present study, we are dealing with amorphous materials, and direct referencing might not be very appropriate. However, one can suppose that the difference between Sens${}_{NR}$ and Sens${}_{AR}$ observed for NR and AR ribbons may have a contribution related to the difference of the stress distribution in the elements with different pre-history and, therefore, providing distinct types of thermal conductivity in different directions. In this regard, new types of thermoelectric composites consisting of rapidly quenched magnetic ribbons capped by thin films deposited by sputtering might be of special interest to study in the future. One of the other important points for thermomagnetic applications could be thermal stability in the field of the temperatures under consideration. Usually, amorphous ribbons in the initial state have a higher level of internal stress and a higher relaxation response in the course of heating. This means that stress-annealed ribbons with lower Sens${}_{AR}$ coefficient may have preferable functional characteristics.

## 4. Conclusions

The structural, quasi-static magnetic properties and thermomagnetic behavior of as-quenched and stress-annealed CoFeCrSiB rapidly quenched amorphous ribbons were studied. The magnetic properties correspond to soft magnetic behavior, with high magnetic permeability and remarkable linearity of magnetization dependence on the value of the applied magnetic field in the low field range. The ANE measurements, carried out in a wide range of magnetic fields and temperature gradients, reflect the magnetic results, presenting a linear response in the thermomagnetic signal, an interesting feature for sensor applications. Irrespective of the studied system (as quenched or annealed sample), the ANE response presents a strong dependence on the magnetic field intensity and direction, which make suitable the use of this system as a magnetic sensor. From the ANE results, we reached $S_{ANE}\left(NR\right)=2.66\;\mu$V/K and $S_{ANE}\left(AR\right)=1.93\;\mu$V/K, for the NR and AR ribbons, respectively. Calculated sensitivities of Sens${}_{NR}=0.33\;\mu$V/Oe and Sens${}_{AR}=0.22\;\mu$V/Oe reach promising application values of the ANE coefficient and thermomagnetic sensitivity for all studied cases. At last, it is worth mentioning that the studied flexible Co-based ribbon can be explored to probe curved surfaces and be used to generate green energy from the heat dissipation of surfaces. 

## Data Availability

The data that support the findings of this study are available from the corresponding author upon reasonable request.
